# Supplementary material for: Content-rich biological network constructed by mining PubMed abstracts
Source: BMC Bioinformatics. 2004 Oct 8;5:147. doi: 10.1186/1471-2105-5-147 (PMC528731; doi:10.1186/1471-2105-5-147)
Supplement: Additional File 2 — The original results of the above study (non-essential files are deleted to keep the file size under the limit set by BMC bioinformatics). [file 1471-2105-5-147-S2.bz2 › chilibotAdditionalFile2/dip50/747ID9150216E18129/html/EXBB_TONB.html]

 


 **EXBB** and **TONB** 
  
Found 83 abstracts in PubMed, retrieved 50.  
 

 What does Google say? 
 PDF only 
| .edu only 

---

**Interactive relationship** (e.g. stimulation, inhibition, etc)

**Neutral relationship**- The amino terminal signal anchor of  **TonB**  and its interaction with the cytoplasmic membrane protein  **ExbB**  are essential to this process.  Ref: 11087740 J Biol Chem, 2001
- The cytoplasmic membrane proteins  **ExbB**  and ExbD support  **TonB**  dependent active transport of iron siderophores and vitamin B12 across the essentially unenergized outer membrane of Escherichia coli.  Ref: 9811664 J Bacteriol, 1998
- The  **tonB**  Bb gene was situated upstream of  **exbB**  and exbD homologues and downstream of a putative Fur regulated promoter.  Ref: 10517598 Microbiology, 1999
- **ExbB**  and ExbD proteins are part of the  **TonB**  dependent energy transduction system and are encoded by the exb operon in Escherichia coli.  Ref: 12193634 J Bacteriol, 2002
- Both  **ExbB**  and ExbD were also required for association of  **TonB**  with the cytoplasmic membrane.  Ref: 12193634 J Bacteriol, 2002
- A second  **tonB**  gene in Pseudomonas aeruginosa is linked to the  **exbB**  and exbD genes.  Ref: 10689178 FEMS Microbiol Lett, 2000
- Energy coupled transport across the outer membrane of Escherichia coli  **ExbB**  binds ExbD and  **TonB**  in vitro, and leucine 132 in the periplasmic region and aspartate 25 in the transmembrane region are important for ExbD activity.  Ref: 8631671 J Bacteriol, 1996
- These results were consistent with the idea that  **ExbB**  and ExbD play roles in the assembly of  **TonB**  into an energy transducing complex.  Ref: 7642501 J Bacteriol, 1995
- TetA  **TonB**  protein, which cannot interact with  **ExbB**  D, was likewise found associated with the outer membrane.  Ref: 9159515 Mol Microbiol, 1997
- These results indicated that the role of  **ExbB**  D in energy transduction is to bring  **TonB**  that has reached the outer membrane back to associate with the cytoplasmic membrane.  Ref: 9159515 Mol Microbiol, 1997
- Conserved residues Ser 16 and His 20 and their relative positioning are essential for  **TonB**  activity, cross linking of  **TonB**  with  **ExbB** , and the ability of  **TonB**  to respond to proton motive force.  Ref: 11087740 J Biol Chem, 2001
- Deletion of Trp 11 resulted in a  **TonB**  that remained active yet was unable to cross link with  **ExbB** .  Ref: 11087740 J Biol Chem, 2001
- Specifically, both  **ExbB**  and ExbD were found to be required for  **TonB**  to conformationally respond to proton motive force.  Ref: 12193634 J Bacteriol, 2002
- This genetic organization has been reported in Haemophilus influenzae, H. ducreyi, Pseudomonas putida and Vibrio cholerae, and may represent a separate lineage of evolution from that of the Enterobacteriaceae in which  **tonB**  is unlinked with the accessory genes  **exbB**  and exbD.  Ref: 9074497 Gene, 1997
- In this paper we show that ExbD protein, encoded by an adjacent gene in the exb cluster at 65, was also required for  **TonB**  dependent energy transduction and, like  **ExbB** , was required for the stability of  **TonB** .  Ref: 7642501 J Bacteriol, 1995
- both were eluted with the  **ExbB**  labeled with 6 histidine residues, demonstrating interaction of  **ExbB**  with ExbD and  **TonB** .  Ref: 8631671 J Bacteriol, 1996
- Interactions in the  **TonB**  dependent energy transduction complex  **ExbB**  and ExbD form homomultimers.  Ref: 9811664 J Bacteriol, 1998
- Energy for active transport is obtained from the proton motive force of the inner membrane through physical interaction with  **TonB**   **ExbB**  ExbD, an inner membrane complex.  Ref: 9886293 Nat Struct Biol, 1999
- It is proposed that the  **TonB**   **ExbB**  ExbD complex opens via an energized conformation of the  **TonB**  protein channels in the outer membrane, formed by proteins which serves as highly specific binding sites for the various ferric siderophores and vitamin B12.  Ref: 7654405 FEMS Microbiol Rev, 1995
- These data further support the concept that  **TonB** ,  **ExbB** , and ExbD form a complex in which the energized conformation of  **TonB**  opens the channels in the outer membrane receptor proteins.  Ref: 8631671 J Bacteriol, 1996
- The aim of this review is to describe an in vivo assay of the interactions taking place in the Tol Pal or  **TonB**   **ExbB**  ExbD envelope complexes in the periplasm of Escherichia coli and between them and colicins or g3p protein of filamentous bacteriophages.  Ref: 12423784 Biochimie< MedlineTA>Biochimie, 1996
- Because Trp 11 was demonstrably not involved in the actual cross linking, these results suggest that the  **TonB**   **ExbB**  interaction detected by cross linking occurred at a step in the energy transduction cycle distinct from the coupling of  **TonB**  to the electrochemical gradient.  Ref: 11087740 J Biol Chem, 2001
- Each suppressor  **ExbB**  restored at least partial function to the  **TonB**  mutants, although  **TonB**  delta V17, for which both the conserved motif and the register of the predicted transmembrane domain alpha helix are affected, was the most refractory.  Ref: 10209752 Mol Microbiol, 1999
- Here, two new mutations of the conserved  **TonB**  transmembrane domain SHLS motif were isolated,  **TonB**  S16L and H20Y, as were two new suppressors,  **ExbB**  V35E and V36D.  Ref: 10209752 Mol Microbiol, 1999
- This second defect was evident only after suppression by the  **ExbB**  mutants, which allow transmembrane domain mutants to be energized, and presented as the rapid turnover of  **TonB**  delta V17.  Ref: 10209752 Mol Microbiol, 1999

**Non-interactive relationship** (e.g. studied together, co-existance, homology, etc.)

- Evidence that the absence of  **ExbB**  or ExbD differentially affected the half lives of newly synthesized and steady state  **TonB**  was obtained.  Ref: 7642501 J Bacteriol, 1995
- In addition, we found that transport across the outer membrane via FhuA,  **TonB** ,  **ExbB** , D rather than transport across the cytoplasmic membrane via FhuC, D, B was rate limiting.  Ref: 9613584 Mol Gen Genet, 1998
- Previous studies of a  **TonB**  transmembrane domain mutant  **TonB**  delta V17 and its phenotypical suppressor  **ExbB**  A39E suggested that  **TonB**  is conformationally sensitive.  Ref: 10209752 Mol Microbiol, 1999
- In this study, we report the per cell numbers of  **TonB** ,  **ExbB** , ExbD and FepA for cells grown under iron replete and iron limited conditions.  Ref: 11967085 Mol Microbiol, 2002
- Cloning and functional characterization of Neisseria gonorrhoeae  **tonB** ,  **exbB**  and exbD genes.  Ref: 9140974 Mol Microbiol, 1997
- Transcription initiation of the fec transport genes required the Ton system, consisting of  **TonB** ,  **ExbB** , and ExbD, and was inhibited by carbonylcyanide m chlorophenylhydrazone CCCP and dinitrophenol DNP, which dissipate the electrochemical potential of the cytoplasmic membrane.  Ref: 9044267 Mol Microbiol, 1997
- The  **tonB** ,  **exbB**  and exbD1 genes of Xanthomonas campestris pv.  Ref: 10832632 Microbiology, 2000
- Thus, the sequence comparison identified the ORF1, ORF2 and ORF3 as gonococcal homologues of the E. coli  **tonB** ,  **exbB**  and exbD genes.  Ref: 9140974 Mol Microbiol, 1997
- A comparison of the DNA flanking the  **exbB**  exbD  **tonB**  locus in P. haemolytica A1 and H. influenzae showed that the flanking regions are completely different between the two organisms.  Ref: 9074497 Gene, 1997
- Rather, FecA and the  **TonB** ,  **ExbB**  and ExbD proteins are involved in induction of the fec transport genes independent of their role in ferric citrate transport across the outer membrane.  Ref: 7729419 EMBO J, 1995
- Cross linking analysis of epitope tagged  **ExbB**  and ExbD proteins with  **TonB**  antisera afforded detection of a previously unrecognized  **TonB**  ExbD cross linked complex and confirmed the composition of the  **TonB**   **ExbB**  cross linked complex.  Ref: 9811664 J Bacteriol, 1998
- By mutational analysis, the  **tonB** ,  **exbB** , and exbD1 genes were shown to be essential for ferric iron import in X. campestris pv.  Ref: 9371459 J Bacteriol, 1997
- Based on sequence similarities, the Bordetella pertussis  **tonB**   **exbB**  exbD locus was identified on a cloned DNA fragment.  Ref: 10722583 Infect Immun, 2000
- In gram negative bacteria, high affinity iron uptake requires the  **TonB**   **ExbB**  ExbD envelope complex to release iron chelates from their specific outer membrane receptors into the periplasm.  Ref: 10722583 Infect Immun, 2000
- Protonmotive force,  **ExbB**  and ligand bound FepA drive conformational changes in  **TonB** .  Ref: 10209752 Mol Microbiol, 1999
- The exbD2 gene as well as the iron uptake genes  **tonB** ,  **exbB**  and exbD1 of Xanthomonas campestris pv.  Ref: 10832632 Microbiology, 2000
- Analysis of sequence alignments of TolQ and TolR homologues from numerous Gram negative bacterial genomes, together with analyses of the different tolQ tolR mutants, revealed that the TM domains of TolQ and TolR present structural and functional homologies not only to  **ExbB**  and ExbD of the  **TonB**  system but also with MotA and MotB of the flagellar motor.  Ref: 11722743 Mol Microbiol, 2001
- In gram negative organisms, high affinity transport of iron substrates requires energy transduction to specific outer membrane receptors by the  **TonB**   **ExbB**  ExbD complex.  Ref: 12562789 J Bacteriol, 2003
- Pasteurella multocida  **exbB** , exbD and  **tonB**  genes are physically linked but independently transcribed.  Ref: 12044675 FEMS Microbiol Lett, 2002
- Neisseria meningitidis  **tonB** ,  **exbB** , and exbD genes Ton dependent utilization of protein bound iron in Neisseriae.  Ref: 9006036 J Bacteriol, 1997
- Uptake of the mutated colicin U DTMVV depended on  **TonB**  and  **ExbB**  and still remained TolA and TolQ dependent, but no longer required TolB.  Ref: 9682486 FEMS Microbiol Lett, 1998
- Reverse transcription RT PCR, using RNA from organisms grown under iron replete and iron restricted conditions, revealed that  **tonB** ,  **exbB** , exbD, tbpB and tbpA are transcribed as a single unit with expression being up regulated in response to iron restriction.  Ref: 12742718 Vet Microbiol, 2003
- group B colicins use the proteins  **TonB** ,  **ExbB** , and ExbD.  Ref: 7545150 J Bacteriol, 1995
- The Ton system consisting of the proteins  **TonB** ,  **ExbB**  and ExbD is required for colicin and microcin import.  Ref: 12423780 Biochimie< MedlineTA>Biochimie, 1995
- Transport of heme by the ShuA protein requires  **TonB**  and its accessory proteins  **ExbB**  and ExbD.  Ref: 9393841 Infect Immun, 1997
- Vibrio cholerae iron transport haem transport genes are linked to one of two sets of  **tonB** ,  **exbB** , exbD genes.  Ref: 9781885 Mol Microbiol, 1998
- It is postulated that FecA,  **TonB** ,  **ExbB**  and ExbD transfer the signal across the outer membrane, while the regulatory protein FecR transmits the signal across the cytoplasmic membrane to FecI in the cytoplasm.  Ref: 7729419 EMBO J, 1995
- In addition, outer membrane receptors together with the  **TonB**   **ExbB**  ExbD complex are directly involved in induction of the transcription of ferric citrate and pseudobactin transport genes of Escherichia coli and Pseudomonas putida, respectively.  Ref: 7654405 FEMS Microbiol Rev, 1995
- , Triton X 100 TX 100 did not require the  **TonB** ,  **ExbB** , or ExbD protein.  Ref: 11796379 Antimicrob Agents Chemother, 2002
- The energy of the proton motive force is postulated to be transduced to the transport proteins by a protein complex that consists of the  **TonB** ,  **ExbB** , and ExbD proteins.  Ref: 11566987 J Bacteriol, 2001
- Mutational analysis revealed that the ferric iron uptake genes  **tonB** ,  **exbB**  and exbD1 are necessary for the induction of a hypersensitive response HR on the nonhost plant pepper Capsicum annuum and the induction of typical black rot symptoms on the host plant cauliflower Brassica oleracea.  Ref: 10832632 Microbiology, 2000
- The electrochemical potential of the cytoplasmic membrane CM energizes transport across the outer membrane, which requires an energy transducing device, consisting of the proteins  **TonB** ,  **ExbB**  and ExbD, from the CM to the OM.  Ref: 9377472 Biol Chem, 1997
- Other biochemical evidence also shows that the proteins that provide energy for iron transport at the outer membrane, such as the  **TonB**   **ExbB**  ExbD system, are structurally very similar across bacterial species.  Ref: 11895294 Curr Top Med Chem, 2001
- In the absence of  **ExbB**  or ExbD, newly synthesized  **TonB**  was degraded with a half life of 5 to 10 min, while the half life of  **TonB**  under steady state conditions was significantly longer, approximately 30 min.  Ref: 7642501 J Bacteriol, 1995
- Quantification of known components of the Escherichia coli  **TonB**  energy transduction system  **TonB** ,  **ExbB** , ExbD and FepA.  Ref: 11967085 Mol Microbiol, 2002
- Analysis of the nucleotide sequence in the complementing DNA showed that it codes for three genes in the order of  **exbB**  exbD  **tonB** .  Ref: 9074497 Gene, 1997
- Cloning and characterization of the  **exbB**  exbD  **tonB**  locus of Pasteurella haemolytica A1.  Ref: 9074497 Gene, 1997
- Characterization of the exbBD operon of Escherichia coli and the role of  **ExbB**  and ExbD in  **TonB**  function and stability.  Ref: 7642501 J Bacteriol, 1995
- Due to the low iron concentration in the plant tissue, the titre of viable bacteria of the ferric iron uptake mutants  **tonB** ,  **exbB**  and exbD1 decreased after leaf infiltration of pepper.  Ref: 10832632 Microbiology, 2000
- Results of insertional mutations, DNA and protein sequence analyses, phage sensitivity tests, transfection tests, complementation tests, and phage adsorption assays together with the cellular location of the proteins indicate that  **TonB** ,  **ExbB** , and ExbD1 are essential for penetration of phage phi L7.  Ref: 12646254 Biochem Biophys Res Commun, 2003
- Transport of bound chelates across the outer membrane depends upon  **TonB**   **ExbB**  ExbD, a cytoplasmic membrane localized complex that transduces energy from the proton motive force to high affinity receptors in the outer membrane.  Ref: 9643536 Mol Microbiol, 1998
- Both full length  **TonB**  and truncated  **TonB**  consisting of the amino terminal two thirds achieved identical conformations in the cytoplasmic membrane, as determined by their abilities to cross link to the cytoplasmic membrane protein  **ExbB**  and their abilities to respond conformationally to the presence or absence of proton motive force.  Ref: 12823822 Mol Microbiol, 2003
- The reconstitution in Escherichia coli of the S. marcescens Has system demonstrated that haem uptake is dependent on the E. coli  **ExbB** , ExbD and  **TonB**  proteins.  Ref: 11737642 Mol Microbiol, 2001
- Following incubation on media containing hemoglobin, N. meningitidis IR3436  **tonB**   **exbB**  exbD deletion mutant and N. gonorrhoeae PD3401  **tonB**  insertional mutant give rise to colonies which can grow with hemoglobin.  Ref: 10986265 J Bacteriol, 2000
- Energy from the cytoplasmic membrane is transferred to the outer membrane with the aid of the Ton system, consisting of the proteins  **TonB** ,  **ExbB** , and ExbD.  Ref: 8631671 J Bacteriol, 1996
- A complex of three membrane spanning proteins  **TonB** ,  **ExbB**  and ExbD couples the chemiosmotic potential of the cytoplasmic membrane with siderophore uptake across the outer membrane.  Ref: 12409204 Biochim Biophys Acta, 2002
- A putative Fur binding sequence located upstream from  **tonB**  was detected in a Fur titration assay, indicating that the  **tonB**   **exbB**  exbD operon may be Fur repressed in high iron growth conditions.  Ref: 10722583 Infect Immun, 2000
- Nevertheless, the  **tonB** ,  **exbB**  and exbD1 mutant strains were able to spread systemically in cauliflower.  Ref: 10832632 Microbiology, 2000
- Sequencing of the Ton system genes revealed that the arrangement of the genes was  **exbB**  exbD  **tonB** .  Ref: 9423852 Infect Immun, 1998
- The  **exbB** , exbD and  **tonB**  genes of the Pasteurella multocida animal pathogen have been cloned by complementation of an Escherichia coli  **tonB**  mutant.  Ref: 12044675 FEMS Microbiol Lett, 2002
- campestris revealed four open reading frames which, based on sequence homologies, were designated  **tonB** ,  **exbB** , exbD1, and exbD2.  Ref: 9371459 J Bacteriol, 1997
- Crystal structures of two outer membrane transport proteins, FhuA and FepA, and biochemical data reveal strong long range conformational changes of the proteins upon binding of Fe 3 siderophore complexes and in response to energy transfer from the cytoplasmic membrane into the outer membrane via the  **TonB**   **ExbB**  ExbD protein complex.  Ref: 11934617 Curr Opin Microbiol, 2002
- However, inducible levels of TX 100 resistance in gonococci had an absolute requirement for the  **TonB**   **ExbB**  ExbD system, suggesting that such resistance in gonococci has an energy requirement above and beyond that required for constitutive pump activity.  Ref: 11796379 Antimicrob Agents Chemother, 2002
- **TonB** , the energy transducer, appears to go through a cycle during energy transduction, with the absence of both  **ExbB**  and ExbD creating blocks at two points i in the inability of  **TonB**  to respond to the cytoplasmic membrane proton motive force and ii in the conversion of  **TonB**  from a high affinity outer membrane association to a high affinity cytoplasmic membrane association.  Ref: 12193634 J Bacteriol, 2002
- PCR approaches allowed the amplification, cloning and sequencing of A. suis  **tonB** ,  **exbB** , exbD, tbpB and tbpA homologues.  Ref: 12742718 Vet Microbiol, 2003
- In Gram negative bacteria, the  **TonB**   **ExbB**  ExbD inner membrane multiprotein complex is required for active transport of diverse molecules through the outer membrane.  Ref: 11737642 Mol Microbiol, 2001
- The ratio of these proteins was calculated at one  **TonB**  two ExbD seven  **ExbB**  under all four growth conditions tested.  Ref: 11967085 Mol Microbiol, 2002
- **ExbB**  and ExbD each formed two unique cross linked complexes which were not dependent on the presence of  **TonB** , the outer membrane receptor protein FepA, or the other Exb protein.  Ref: 9811664 J Bacteriol, 1998
- Allelic replacement of the wild type H. ducreyi  **exbB** , exbD, and  **tonB**  loci with the  **exbB** , exbD, and  **tonB**  deletion resulted in an H. ducreyi isogenic mutant unable to utilize hemoglobin but able to utilize hemin at the same levels as the parent strain to fulfill its heme requirement.  Ref: 9423852 Infect Immun, 1998
- Energy flows from the cytoplasmic into the outer membrane via a complex consisting of the  **TonB** ,  **ExbB**  and ExbD proteins which are anchored in the cytoplasmic membrane.  Ref: 7654405 FEMS Microbiol Rev, 1995
- Nucleotide sequence analysis of DNA fragments subcloned from the Hb complementing cosmids identified four open reading frames, three of them homologous to Pseudomonas putida, E. coli, and Haemophilus influenzae  **exbB** , exbD, and  **tonB**  genes.  Ref: 9006036 J Bacteriol, 1997
- As demonstrated previously,  **TonB**  can undergo at least one conformational change, provided both  **ExbB**  and a functional  **TonB**  transmembrane domain are present.  Ref: 10209752 Mol Microbiol, 1999
- They share homology with the  **ExbB** , ExbD, and  **TonB**  proteins, respectively.  Ref: 11418549 J Bacteriol, 2001
- The signal elicited by ferric citrate crosses the outer membrane via  **TonB** ,  **ExbB** , and ExbD.  Ref: 8602163 Mol Gen Genet, 1996
- The energy is transduced to FecA by the inner membrane complex,  **TonB** ,  **ExbB** , and ExbD.  Ref: 12618451 J Bacteriol, 2003
- It is thought that the proteins  **TonB** ,  **ExbB** , and ExbD function as a coupling device between the cytoplasmic membrane and the outer membrane.  Ref: 9457864 J Bacteriol, 1998
- This gene is clustered with three other genes,  **tonB**   **exbB**  exbD1 exbD2.  Ref: 12646254 Biochem Biophys Res Commun, 2003
- Uptake of colicin Js into sensitive bacteria was dependent on the  **ExbB**  protein but not on the E. coli K 12  **TonB**  and TolA, B, and Q proteins.  Ref: 11395459 J Bacteriol, 2001
- Inducible, but not constitutive, resistance of gonococci to hydrophobic agents due to the MtrC MtrD MtrE efflux pump requires  **TonB**   **ExbB**  ExbD proteins.  Ref: 11796379 Antimicrob Agents Chemother, 2002
- **ExbB**  and ExbD do not function independently in  **TonB**  dependent energy transduction.  Ref: 12193634 J Bacteriol, 2002
- Vibrio cholerae was found to have two sets of genes encoding  **TonB** ,  **ExbB**  and ExbD proteins.  Ref: 9781885 Mol Microbiol, 1998
- In this study, Escherichia coli  **TonB**  was found to be distributed in sucrose density gradients approximately equally between the cytoplasmic membrane and the outer membrane fractions, while two proteins with which it is known to interact,  **ExbB**  and ExbD, as well as the NADH oxidase activity characteristic of the cytoplasmic membrane, were localized in the cytoplasmic membrane fraction.  Ref: 9159515 Mol Microbiol, 1997
- When  **ExbB**  and ExbD, as well as their cross talk competent homologues ToIQ and ToIR, were absent,  **TonB**  was found associated with the outer membrane.  Ref: 9159515 Mol Microbiol, 1997
- Analysis of translational fusions to alkaline phosphatase and beta galactosidase confirmed that the  **TonB** ,  **ExbB** , ExbD1, and ExbD2 proteins are anchored in the cytoplasmic membrane.  Ref: 9371459 J Bacteriol, 1997
- Energy coupled transport and signal transduction through the gram negative outer membrane via  **TonB**   **ExbB**  ExbD dependent receptor proteins.  Ref: 7654405 FEMS Microbiol Rev, 1995
- campestris  **tonB** ,  **exbB** , and exbD1 are essential for ferric iron uptake, but exbD2 is not.  Ref: 9371459 J Bacteriol, 1997
